# Genomic and functional analysis of the host response to acute simian varicella infection in the lung

**DOI:** 10.1038/srep34164

**Published:** 2016-09-28

**Authors:** Nicole Arnold, Thomas Girke, Suhas Sureshchandra, Christina Nguyen, Maham Rais, Ilhem Messaoudi

**Affiliations:** 1 Graduate Program in Microbiology, University of California-Riverside, CA, USA; 2 Department of Botany and Plant Sciences, University of California-Riverside, CA, USA; 3 Graduate Program in Genetics, Genomics and Bioinformatics, University of California-Riverside, CA, USA; 4 Division of Biomedical Sciences, School of Medicine, University of California-Riverside, Riverside, CA, USA

## Abstract

Varicella Zoster Virus (VZV) is the causative agent of varicella and herpes zoster. Although it is well established that VZV is transmitted via the respiratory route, the host-pathogen interactions during acute VZV infection in the lungs remain poorly understood due to limited access to clinical samples. To address these gaps in our knowledge, we leveraged a nonhuman primate model of VZV infection where rhesus macaques are intrabronchially challenged with the closely related Simian Varicella Virus (SVV). Acute infection is characterized by immune infiltration of the lung airways, a significant up-regulation of genes involved in antiviral-immunity, and a down-regulation of genes involved in lung development. This is followed by a decrease in viral loads and increased expression of genes associated with cell cycle and tissue repair. These data provide the first characterization of the host response required to control varicella virus replication in the lung and provide insight into mechanisms by which VZV infection can cause lung injury in an immune competent host.

Varicella zoster virus (VZV) is a neurotropic alpha herpes virus and the causative agent of varicella and herpes zoster. VZV is primarily acquired through the inhalation of infectious virus particles or via direct contact with skin lesions^1^. Although varicella is a benign self-limiting disease, adults (especially smokers and pregnant women) and immunocompromised persons frequently develop serious complications, notably varicella pneumonia (VP)^2^,^3^, which can result in acute respiratory distress syndrome (ARDS) in severe cases^4^.

Mechanisms of VZV pathogenesis and the host response to VZV infection in the lungs is poorly understood because of limited access to human lung biopsies during acute infection and the strict host species specificity of VZV. Simian Varicella Virus (SVV) shares 70–75% DNA homology with VZV^5^ and intra-bronchial infection of rhesus macaques (RM) recapitulates the clinical features of primary VZV^6^. Recent studies from our group have reported the presence of a vigorous anti-SVV response characterized by effector memory CD8 T cell infiltration, a robust CD4 T cell response, and production of antiviral cytokines/chemokines and growth factors in the bronchoalveolar lavage (BAL)^7^. However, we still do not have a clear understanding of the host response to varicella virus within the lung tissue. Here, we leveraged this animal model to characterize SVV-induced immune response as well as changes in lung histopathology and gene expression during primary varicella infection. Our results show that SVV infection results in lung injury as evidenced by focal hemorrhaging collapse of airways, and reduced expression of genes with a role in lung development and/or function. SVV infection also induces a robust immune response as evidenced by immune cell infiltration, production of pro-inflammatory cytokines and chemokines, and increased expression of genes involved in host defense. The development of this immune response correlates with decreased SVV viral loads. Moreover, as viral loads decrease, a shift towards increased expression of genes with a role in tissue regeneration and increased levels of growth factors is observed. Collectively, these data increase our understanding of mechanisms of host defense and the development of respiratory complications during VZV infection.

## Results

### SVV infection results in significant lung inflammation

**Figure 1:**
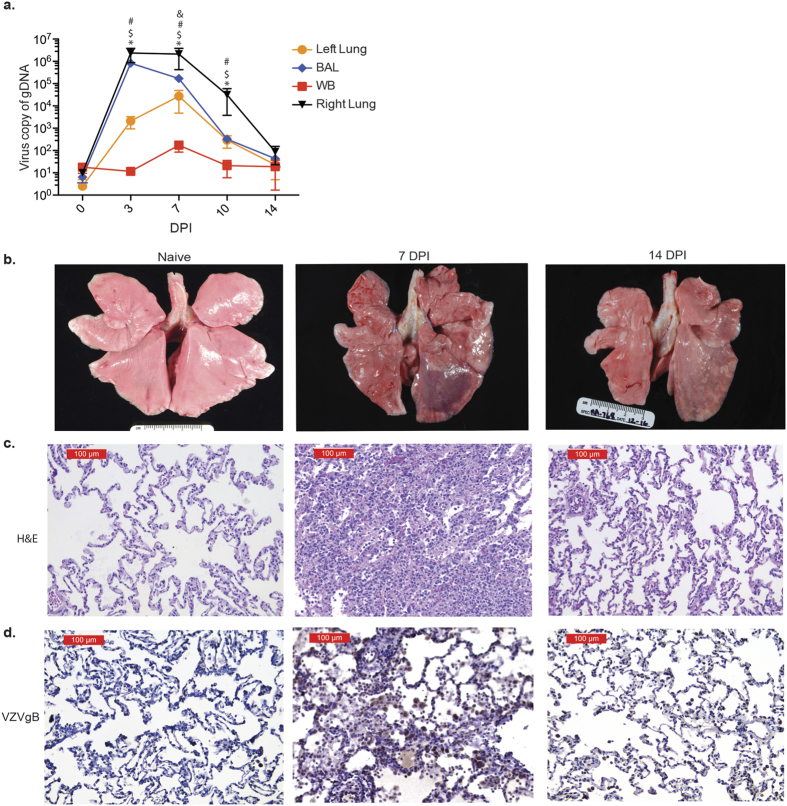
SVV infection results in inflammation. (**a**) SVV viral loads in lung biopsies, bronchial alveolar lavage (BAL) and blood (WB) were measured by quantitative PCR using primers and probes specific for SVV *ORF21* (BAL: n = 14 (0 days post infection, DPI), n = 11 (3 DPI), n = 8 (7 DPI), n = 5 (10 DPI), n = 3 (14 DPI); Lung: n = 3 (0 DPI), n = 3 (3 DPI), n = 3 (7 DPI), n = 2 (10 DPI), n = 3 (14 DPI); WB: n = 14 (0 DPI), n = 11 (3 DPI), n = 8 (7 DPI), n = 5 (10 DPI), n = 3 (14 DPI)) (^#^p < 0.05 for left lung; ^$^p < 0.05 for BAL; ^&^p < 0.05 for WB; *p < 0.05 for right lung relative to day 0). (**b**) SVV infection results in focal hemorrhage during peak viral replication that largely resolved 14 DPI. (**c**) H&E staining shows immune infiltrates and lung consolidation during peak viral replication. (**d**) VZVgB staining showing high levels of viral antigen 7 DPI that were decreased 14 DPI.

Eleven rhesus macaques were intrabronchially inoculated with 4 × 10^5 ^PFU of SVV into their right bronchus and euthanized 3 (n = 3), 7 (n = 3), 10 (n = 2) and 14 (n = 3) days post infection (DPI). Viral loads peaked in the BAL and infected right lung 3 DPI and in the uninfected left lung and blood 7 DPI, followed by cessation of viral replication 14 DPI (Fig. 1a). In line with viral replication kinetics, the lungs showed severe focal hemorrhaging and firm nodules 7 DPI, which resolved 14 DPI (Fig. 1b). Similarly, H&E staining revealed damage to the alveolar walls and perivascular infiltration of lymphocytes and macrophages that peaked 7–10 DPI, indicative of viral pneumonia, which also resolved 14 DPI (Fig. 1c). To determine the association between viral replication and viral antigen, lung tissue slides were stained with anti-VZVgB (which shares 71% amino acid identity with SVVgB). As described for viral DNA loads, VZVgB staining peaked 7 DPI (Fig. 1d).

**Figure 2:**
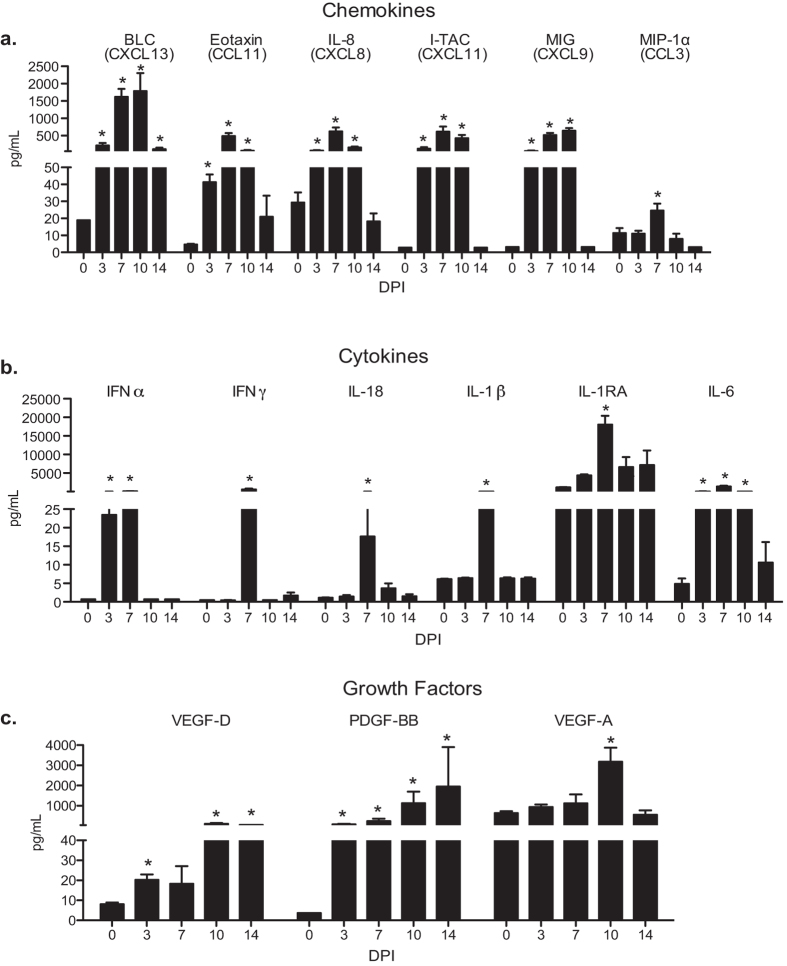
SVV infection induces secretion of cytokines, chemokines and growth factors. Levels of (**a**) chemokine, (**b**) cytokines and (**c**) growth factors in the BAL were measured using Luminex technology. BAL: n = 14 (0 days post infection, DPI), n = 11 (3 DPI), n = 8 (7 DPI), n = 5 (10 DPI), n = 3 (14 DPI) Mean pg/ml ± SEM (*p < 0.05 compared to day 0).

The concentration of chemokines, cytokines and growth factors in BAL supernatants and lung homogenates were measured using Luminex technology. Levels of the B cell chemoattractant BLC, eosinophil attractant eotaxin, granulocyte chemoattractants IL-8 and MIP1α, as well as T cell chemokines I-TAC and MIG increased 3–10 DPI (Fig. 2a). Levels of IL-6, which plays a critical role in viral clearance, also increased 3–10 DPI (Fig. 2b). In addition, levels of the potent antiviral cytokine IFNα were increased 3 and 7 DPI (Fig. 2b), while levels of IFNγ, IL-18 (induces IFNγ production by T cells), IL-1β, and its antagonist IL-1RA were increased only 7 DPI (Fig. 2b). Moreover, levels of growth factors VEGF-D and PDGF-BB were increased 3–14 DPI and those of the pro-angiogenic factor VEGF-A were increased 10 dpi (Fig. 2c). In contrast, the lung homogenates showed no significant change in protein levels of chemokines, cytokines or growth factors.

### SVV infection leads to the recruitment of T cells and phagocytes

**Figure 3:**
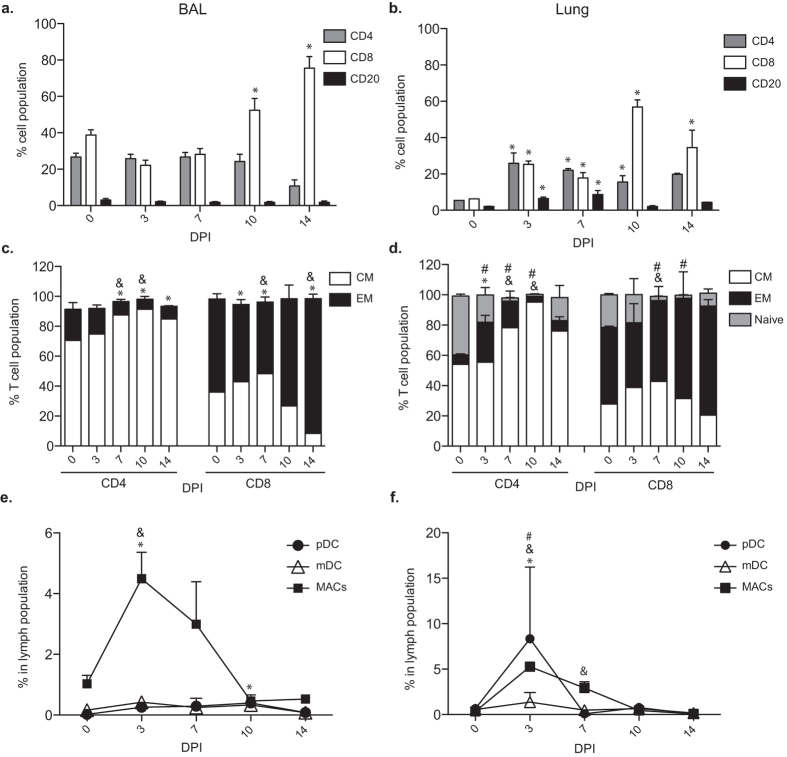
SVV infection induces immune infiltration. (**a,b**) The frequencies (means ± SEM) of CD4, CD8 and CD20 positive cells in (**a**) BAL and (**b**) the lungs (*p < 0.05 compared to day 0). (**c,d**) The percentage of naïve, central memory (CM) and effector memory (EM) CD4 and CD8 T cells in the (**c**) BAL (^&^*p* < 0.05 for CM; *p < 0.05 for EM compared to day 0) and (**d**) lungs (^&^p < 0.05 for CM; *p < 0.05 for EM; ^#^p < 0.05 for naïve compared to day 0). (**e,f**) The percentage of plasmacytoid DCs (pDCs), myeloid DCs (mDCs), and macrophages (MACs) in (**e**) BAL and (**f**) lungs (^&^p < 0.05 for MACs; *p < 0.05 for pDCs; ^#^p < 0.05 for mDCs). BAL: n = 14 (0 days post infection, DPI), n = 11 (3 DPI), n = 8 (7 DPI), n = 5 (10 DPI), n = 3 (14 DPI); Lung: n = 3 (0 DPI), n = 3 (3 DPI), n = 3 (7 DPI), n = 2 (10 DPI), n = 3 (14 DPI). Tissues used were from the infected right lobe.

Given the increased concentration of T cell, B cell and granulocyte chemokines in the BAL, we next characterized the immune cells that infiltrated the lungs during acute varicella (Fig. 3). As we previously reported^7^, CD20 cells were rare whereas CD4 and CD8 T cells were abundant pre-infection in BAL. After SVV infection, the frequency of CD8 T cells in the BAL increased 10 and 14 DPI (Fig. 3a). In contrast, frequencies of both B and T cells were low in naïve lungs, but increased as early as 3 DPI, with CD8 T cells showing the largest increase 10 and 14 DPI (Fig. 3b). T cell activation was indicated by the increased prevalence of the highly differentiated CD8 EM T cells and CD4 CM T cells 7 to 14 DPI and CD4 EM 7–10 DPI (Fig. 3c,d). We also observed an increased frequency of plasmacytoid dendritic cells (pDCs), myeloid dendritic cells (mDCs), and macrophages (MACs) 3–7 DPI in both BAL and lung (Fig. 3e,f).

**Figure 4:**
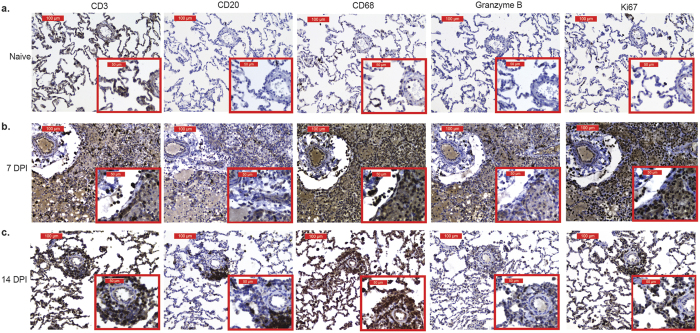
Immune cells proliferate and exhibit cytotoxicity in SVV infected lungs. CD3, CD20, CD68, granzyme B and Ki67 staining in lung sections from (**a**) naïve, (**b**) 7 DPI and (**c**) 14 DPI at 20X and 40X magnification.

To further characterize the immune infiltrates, immunohistochemistry (IHC) was used to determine changes in distribution and frequency of CD3, CD20, CD68, Ki67 and granzyme B (GRZMB) positive cells (Fig. 4). As detected by flow cytometry (Fig. 3), intensity of CD3, CD20 and CD68 staining increased 3 DPI and peaked 7 DPI (Fig. 4b). Peak staining of GRZMB and Ki67 was also observed at 7 DPI and correlated with increased frequency of memory T cells detected by flow cytometry (Fig. 4b). By 14 DPI, CD20 and granzyme B staining was substantially reduced, while CD3, CD68, and Ki67 expression remained higher than baseline (Fig. 4c).

### SVV infection induces robust changes in gene expression

**Figure 5:**
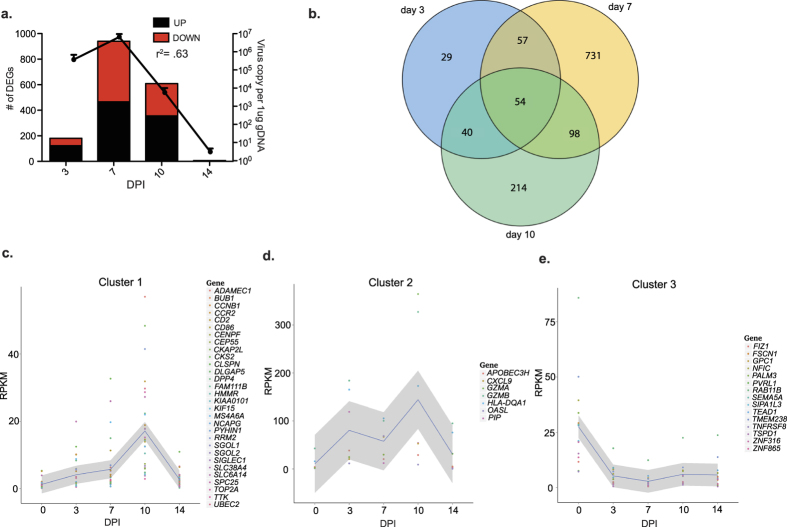
SVV infection results in robust changes in lung gene expression. (**a**) The number of DEGs correlates with viral loads. (**b**) Venn diagram of DEGs detected 3, 7 and 10 DPI. (**c–e**) Gene clusters of the 52 common DEGs with human homologs.

RNA-Seq was used to characterize the host gene expression changes in the lungs during acute varicella. The number of differentially expressed genes (DEGs) correlated tightly with viral loads, with the highest number of DEGs detected 7 DPI (Fig. 5a and Supplemental Datasets S1–4). Changes in gene expression were validated for a subset of genes using qRT-PCR (Supplemental Fig. S1). The 54 DEGs that were differentially expressed 3, 7 and 10 DPI (Fig. 5b) segregated into 3 clusters. Cluster 1 contained 30 DEGs whose expression gradually increased until it peaked 10 DPI (Fig. 5c). Several of these genes are involved in cell cycle such as cyclin kinase subunit 2 (*CKS2*), Ribonucleoside-diphosphate reductase subunit M2 (*RRM2*), and topoisomerase II alpha (*TOP2A*). Cluster 2 contained 7 DEGs whose expression was substantially increased as early as 3 DPI and remained high until 10 DPI before decreasing back to baseline 14 DPI (Fig. 5d). Several of these DEGs play a role in antiviral defense such as granzyme A (*GZMA*), *GZMB*, 2′-5′-Oligoadenylate Synthetase-Like (*OASL*), and *CXCL9*. Cluster 3 contained 15 genes that remained down-regulated throughout acute infection (Fig. 5e). These genes are involved in several cellular processes such as migration (fascin actin-bundling protein 1, *FSCN1*), division (Glypican 1, *GPC1*), and tight junction organization (nectin-1, *PVRL1*, an alpha herpesvirus entry mediator^8^). To gain a better understanding of the biological implications of the gene expression changes we detected, functional enrichment of DEGs that have human homologs was carried out using Metacore software.

### DEGs 3DPI play a role in innate immunity and lung development

**Figure 6:**
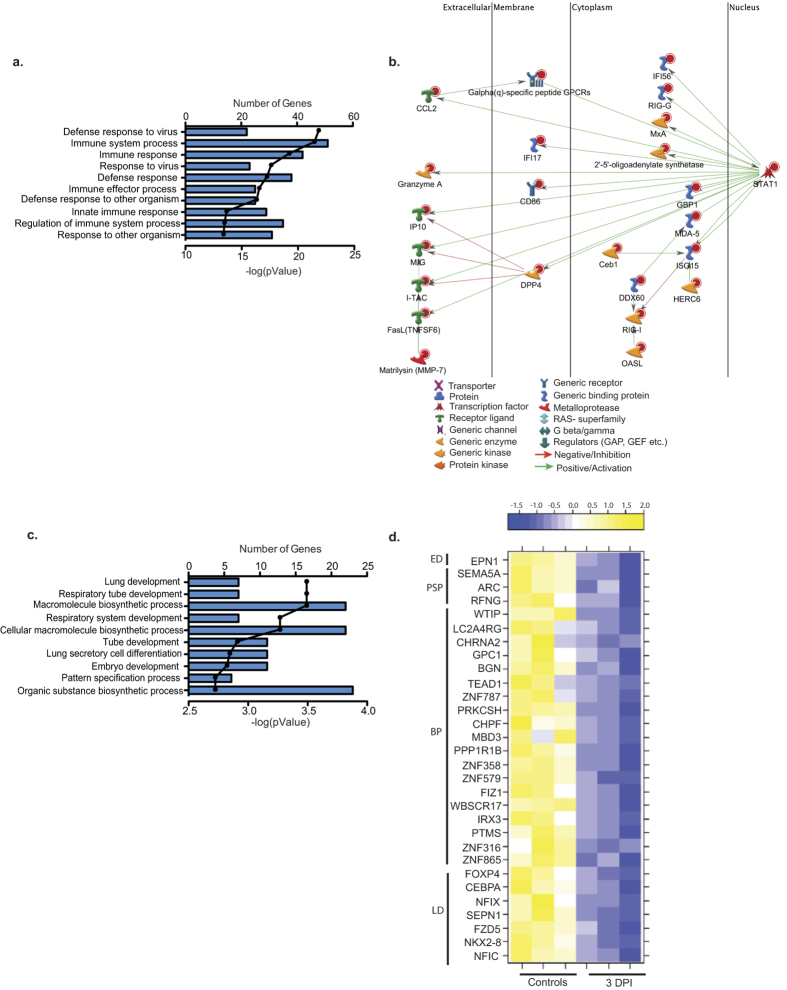
DEGs detected 3 DPI play a role in innate immunity and lung development. (**a**) Bar graph shows the number of genes mapping to each of the 10 most statistically significant GO terms to which the 106 up-regulated genes enriched. Line represents the −log(p-value) associated with each GO term. (**b**) Network of DEGs mapping to the GO process “immune system” that directly interact. (**c**) Bar graph shows the number of genes mapping to each of the 10 most statistically significant GO processes to which the 54 down-regulated genes enriched. Line represents the −log(p-value) associated with each GO term. (**d**) Heat map of the 30 down-regulated DEGs that enriched to the GO terms described in (**c**) grouped by the GO term to which they mapped: ED = Embryo development, PSP = pattern specification process, BP = biosynthetic process and LD = Lung development.

Most of the 106 DEGs that were up-regulated 3 DPI mapped to Gene Ontology (GO) terms associated with antiviral immunity (Fig. 6a). Several genes that enriched to the GO process “immune system process” are part of a network regulated by Signal Transducers and Activators of Transcription 1 (*STAT1*; FC 4) (Fig. 6b). Most of these DEGs have antiviral function such as interferon-stimulated genes (ISGs) *ISG15* (FC 8), *MXA* (FC 7), and *GBP1* (FC 7) as well as dsRNA sensors *DDX60* (FC 5) and *RIG-I* (FC 4)^9^ (Fig. 6b). Other *STAT1* regulated DEGs in our dataset are involved in apoptosis, such as granzyme A (*GZMA*; FC 83), granzyme K (*GZMK*; FC 35), and Fas ligand (*FASL*; FC 12)^10^. Other DEGs play a role in chemotaxis, e.g. *MIG* (*CXCL9*, FC 32), *IP10* (*CXCL10*, FC 17), and *ITAC* (*CXCL11*, FC 14)^11^. Additional highly up-regulated genes include the immune-modulatory prolactin induced protein (*PIP*; FC 51) and lymphocyte differentiation antigen immunoglobulin J *(IGJ*, FC 38).

The 54 down-regulated genes primarily mapped to GO terms associated with lung development (Fig. 6c) such as iroqouis homeobox 3 (*IRX3*; FC7)^12^, nuclear factor I/C (*NFIC*; FC 8)^13^ and WNT frizzled class receptor 5 (*FZD5*; FC8)^14^. The 23 down-regulated DEGs that mapped to biosynthetic pathways play a role in DNA binding and transcriptional regulation, notably zinc finger protein 865 (*ZNF865*; FC 9) and 316 (*ZNF316*; FC 9), and parathymosin (*PTMS*; FC8) (Fig. 6d)^15^,^16^.

Given the immune changes in immune infiltrates into the lung 3 DPI, we wanted to gain a better understanding of which immune cells are expressing these DEGs. To that end, we used the Immunological Genome Project (ImmGen), a database maintained by a collaborative group of immunologists and computational biologists that aim to determine the patterns of gene expression of specific immune cells in the mouse^17^,^18^. This analysis showed that a group of genes that play a role in the cell cycle progression (e.g. *Top2a*, *BUB1*, *CEP55*, *SPC25* and *CENPF*) were highly expressed by B cells, dendritic cells and T cells (Supplemental Fig. S2, cluster 1). We also identified 2 additional clusters of genes that were mainly expressed by MACs (e.g. *ADAMDEC1*, *IgJ, SIGLEC1*, *CXCL9* and *CXCL10*) (Supplemental Fig. S2, cluster 2a,b). Two small clusters are expressed by NK cells (e.g. *GZMB*, *GZMA* and *SAMD3*) (Supplemental Fig. S2 cluster 3a,b), and an additional cluster of antiviral genes was seen in the neutrophils (e.g. DDX60, OAS2, and RASD2) (Supplemental Fig. S2, cluster 4). These data correlate with our flow cytometry analysis (Fig. 3), which report significant increases in T cells, B cells, MACs and DC numbers at 3 DPI in the lung.

In line with our functional analysis, few of the DEGs down-regulated at 3 DPI mapped to immune cell populations. These include *FSCN1* (pathogen recognition molecule^19^) and *ARC* (apoptosis^20^) highly expressed in dendritic cells, and *SEPN1* (oxidation-reduction homeostasis^21^), highly expressed in neutrophils (data not shown).

### DEGs detected 7 DPI primarily map to host defense

**Figure 7:**
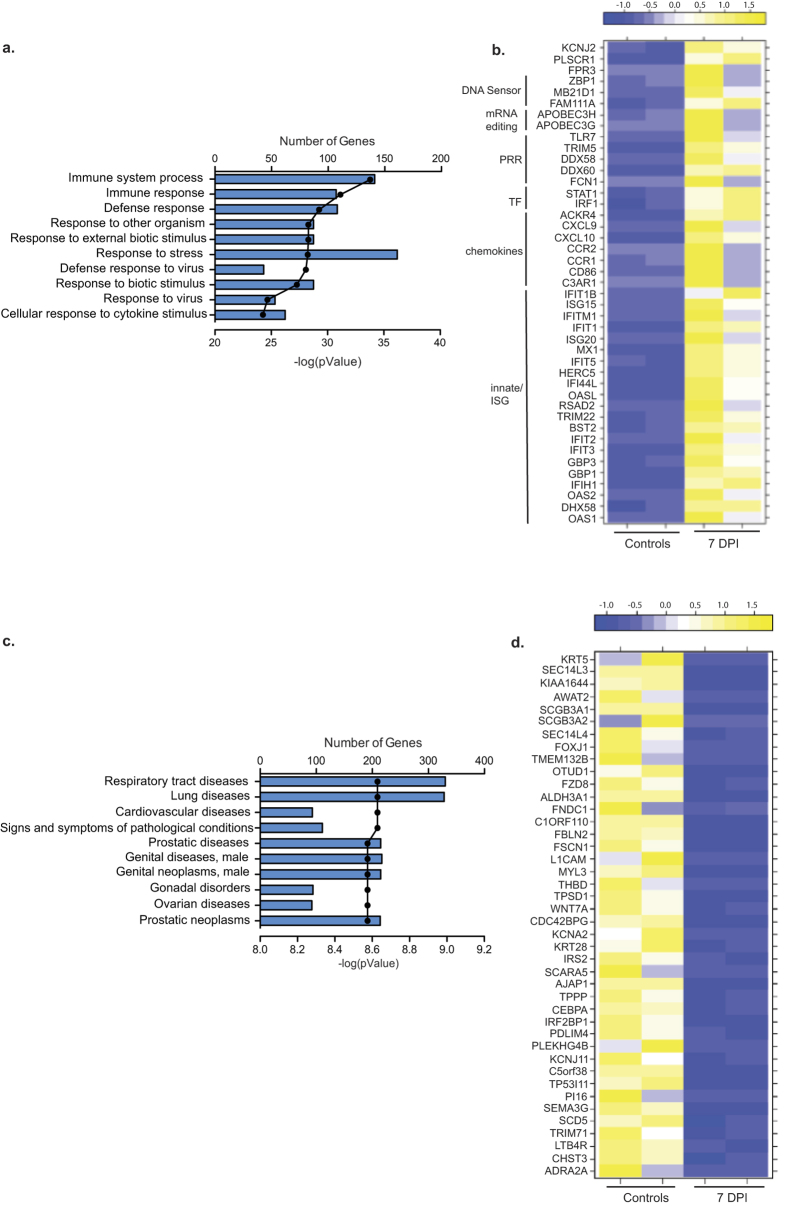
DEGs detected 7 DPI are involved in host defense. (**a**) Bar graph shows the number of genes mapping to each of the 10 most statistically significant GO terms to which the 380 up-regulated genes enriched. Line represents the −log(p-value) associated with each GO term (**b**) Heat map of the genes that mapped to the GO process “response to virus” grouped by function (PRR = pathogen recognition receptor; TF = transcription factor; ISG = immune stimulated gene). (**c**) Bar graph shows the number of genes mapping to each of the 10 most statistically significant disease pathways to which 400 down-regulated genes enriched. Line represents the −log(p-value) associated with each GO term. (**d**) Heat map of the DEGs with a FC >8 that enriched to “respiratory tract diseases” and “lung diseases”.

The 380 up-regulated DEGs detected 7 DPI primarily mapped to GO terms related to host defense (Fig. 7a). Over 100 DEGs involved in anti-viral immunity had a fold change >10 including: *CCL8* (FC 129), *CXCL10* (FC 129), *CCL20* (FC 77), *CCL2* (FC 63), *ISG15* (FC 59), 2′-5′-oligoadenylate synthetase-like (*OASL*, FC 44), *RIGG* (FC 32), *GBP1* (FC 30), and *IL8* (*CXCL8*, FC 28) (Fig. 7b). Additional immune related DEGs that didn’t map to the 10 most significant GO processes include *FAM26F* (FC 92), involved in IFNγ production by NK cells^22^, Pentraxin 3 (*PTX3*, FC 71) involved in complement activation^23^, and tissue inhibitors of metalloproteinases 1 (*TIMP1*, FC 26). Other highly up-regulated DEGs play a role in respiratory diseases such as Hyaluronan synthase 2 (*HAS2*; FC 71) associated with asthma and tissue fibrosis^24^, and metallothionein 2A (*MT2A*, FC 24) involved in lung cancer^25^.

Of the 400 down-regulated DEGs 7 DPI, 300 enriched to GO terms “respiratory tract diseases” and “lung diseases” (Fig. 7c). The most down-regulated gene was keratin 5 (*KRT5*, FC 35), an intermediate filament protein involved in lung regeneration^26^ (Fig. 7d). Down-regulated DEGs with a role in lung function include: insulin receptor substrate 2 (*IRS2*, FC 9), an anti-inflammatory agent in the lung^27^; and *SEC*14-like 3 (*SEC14L3*, FC 33), a lipid packing sensor expressed by alveolar cells and involved in the prevention of lung collapse^28^.

ImmGen analysis of the up-regulated immune genes revealed clusters that are highly expressed by DCs (e.g. *MX1*, *IFIH1*, and *IL-15RA*); MACs (e.g *CCR1*, *CD86* and *IL-1β*); NK cells (*GZMA* and *GZMB*); neutrophils (e.g *OAS2*, *RSAD2* and *TNFSF13B*) and T and B cells (e.g. *RRM2*, *KPNA1* and *EZH2*) (Supplemental Fig. S3, clusters 1–5). We also observed a cluster of inflammatory genes (e.g. *CCL1*, *CXCL1*, *CXCL9*, *CXCL10* and *IFITM1*) that were highly expressed by stromal cells (Supplemental Fig. S3, cluster 6a,b). In line with our functional enrichment, only 1 down-regulated gene (*SEPN1*) was expressed by immune cells (data not shown).

### DEGS at 10 DPI are involved in tissue regeneration

**Figure 8:**
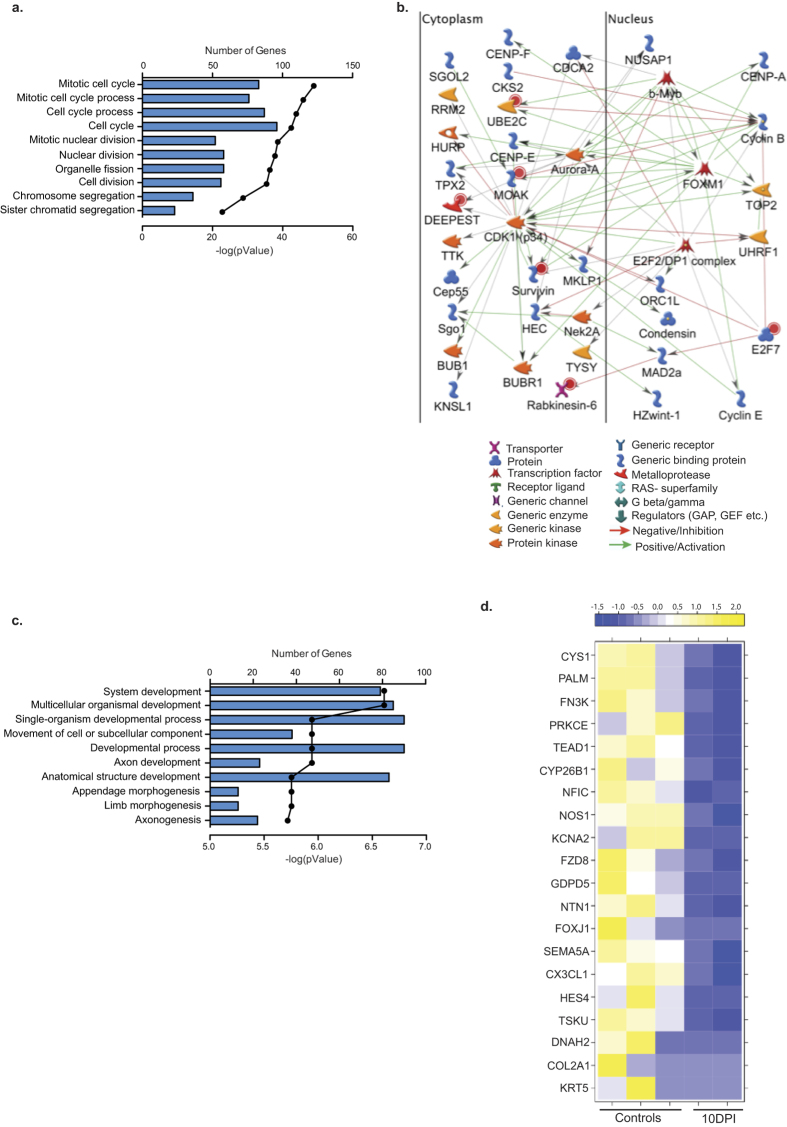
DEGs detected 10 DPI are involved in cell cycle and organ development. (**a**) Bar graph shows the number of genes mapping to each of the 10 most statistically significant GO terms to which the 300 up-regulated genes enriched. Line represents the −log(p-value) associated with each GO term. (**b**) Network image of the 50 most up-regulated genes in the GO term “cell cycle”. (**c**) Bar graph shows the number of genes mapping to each of the 10 most statistically significant GO terms to which the 223 down-regulated genes enriched. Line represents the −log(p-value) associated with each GO term. (**d**) Heat map of the 20 most down-regulated genes found in the GO terms.

At 10 DPI, most of the 300 up-regulated DEGs enriched to GO terms associated with cell cycle (Fig. 8a). Several of these DEGs are part of a network regulated by transcription factors Myb-related protein B (*b-Myb*, FC 9, S- phase^29^) and forkhead box M1 (*FOXM1*, FC 11, S- and M-phase^30^) (Fig. 8b) and modified by serine/threonine kinase, cyclin-dependent kinase 1 (*CDK1*, FC 10, mitosis, G2-M phase transition, apoptosis and genome stability^31^). *CDK1* also interacts with the centrosomal protein 55kDa (*CEP55*, FC 26), involved in cytokinesis^32^; budding uninhibited by benzimidazoles 1 homolog (*BUB1*, FC 26), essential for spindle assembly and chromosome alignment^33^; and ribonucleotide reductase M2 (*RRM2*, FC 22). As noted earlier, several immune related genes identified 7 DPI remained up-regulated 10 DPI (Fig. 5d) including: cytolytic molecules *GZMA* (FC 104), *GZMK* (FC 17), and *GZMB* (FC 8); cytokines/chemokines e.g. interleukin 2 receptor (*IL2RA*, FC 13), *CCR2* (FC 12), and *IL8* (FC 9); and activation markers such as major histocompatibility complex, class II (*HLADQB1*, FC 10) and *IGJ* (FC 12).

The 223 DEGs down-regulated 10 DPI were enriched in GO terms important for organ development (Fig. 8c). The most highly down-regulated genes within these GO processes include *KRT5* (FC 66, lung regeneration^26^), collagen II (*COL2*, FC 30, lung structure^34^), and Netrin-1 (*NTN-1*, FC 6, leukocyte transport into alveolar space^35^) (Fig. 8d). Other highly down-regulated genes include secretoglobin family 1A member 1 (*SCGB1A1*, FC 15, immunomodulation^36^), and troponin T type 1 (*TNNT1*, FC 11 muscle contraction^37^).

ImmGen analysis revealed a large cluster of genes highly expressed in B cells and T cells that play a role in the cell cycle (e.g. *CCNB1*, *BUB1*, and *CDK1*). This enrichment correlates with the significant increase in T cells at 10 DPI in lung tissue (Supplemental Fig. S4, cluster 1, Figs 2a and 3a,b). We also observed a cluster of genes that are highly expressed in MACs (CCR1, CD86, CD163, IgJ, CCL2, and CXCL9); NK and T cells (GZMA, GZMB, CCR2, and CCL5); and T cells (*CD96*, *CD3D* and *CD2*) (Supplemental Fig. 4, clusters 2–4). As described for 3 and 7 DPI, none of the down-regulated DEGs were highly expressed by immune cells (data not shown).

### Gene expression returns to pre-infection levels by 14 DPI

Only 5 DEGs with human homologs were detected 14 DPI. The two up-regulated genes were phospholipase A2 (*PLA2G2A*, FC 37), which is involved in phospholipid metabolism and is overexpressed in lung cancer cells^38^, and solute carrier family 38 member 4 (*SLC38A4*, FC16), important for amino acid transport^39^. The three down-regulated genes played a role in cellular motility: dynein axonemal, heavy chain 2 (*DNAH2*, FC 21), involved in respiratory cilium motility and microtubule movement^40^; protein phosphatase 1 regulatory subunit 1B isoform 1 (*PPP1R1B*, FC 5), which modulates microtubule stability via inhibition of protein-phosphatase 1^41^; and desmin (*DES*, FC 5), a major intermediate filament found in muscle and endothelial cells^42^.

## Discussion

Although VZV is primarily transmitted via inhalation and replicates in the lungs^1^, our understanding of the impact of VZV infection on lung function and the host response to VZV in the respiratory tract remains incomplete. In this study, we leveraged a robust model of VZV infection wherein rhesus macaques are intrabronchially infected with the homologue SVV to carry out the first in depth analysis of the host-pathogen interactions during acute varicella infection in the lungs using a combination of immunohistochemistry, flow cytometry, multiplex ELISA, and RNA sequencing. Our analyses show for the first time that SVV spreads rapidly through the lungs replicating both within the lung parenchyma as well as BAL immune cells. We also report the down-regulation of genes important for lung function, indicative of lung injury during the acute phase of infection. Immune responses and changes in gene expression tightly correlate with viral loads with the up-regulation of pro-inflammatory cytokines, ISGs, chemokines and granzyme genes at peak viral loads. In contrast, genes associated with cell cycle and lung repair were up-regulated after cessation of viral replication.

Immunohistochemistry staining and flow cytometry show that SVV infection results in significant infiltration of lymphocytes and MACs in the lungs as early as 3 DPI and before the appearance of varicella rash, typically detected 10–14 DPI. This is in agreement with previous reports of patients who developed varicella pneumonia before the development of the vesicular rash^3^,^4^. As previously reported for BAL^7^, we saw a higher accumulation of highly differentiated CD8 T cells compared to CD4 T and CD20 B cells within lung biopsies. CD8 T cells were also the most abundant lymphocyte subset in the lungs of mice intra-nasally infected with respiratory syncytial virus (RSV)^43^ and rhesus macaques infected with H1N1 influenza^44^. Furthermore, CD8 T cells were shown to be essential for recovery in mice infected with Vaccinia virus^45^. In addition to lymphocytes, frequency of MACs within the lung biopsy also increased 3 and 7 DPI, returning to pre-infection levels 10 DPI. Bioinformatic analysis of DEGs detected 3–10 DPI using ImmGen also indicate that transcriptional changes are mediated by T cells, B cells, DCs, MACs, neutrophils and NK cells 3 and 7 DPI, with a bigger contribution of DCs, MACs, and neutrophils 7 DPI. In contrast, gene expression changes 10 DPI seem to be primarily originating from lymphocytes.

Concomitant with the influx of T cells, B cells, MACs and DCs into the BAL and lung tissue, we detected a significant increase in T cell attractants I-TAC and MIG, B cell chemokine BLC and granyulocyte attractant IL-8 and eotaxin in the BAL 3 and 7 DPI. Levels of pro-inflammatory cytokines IL-18, IL-1β, IL-6 and IFNγ also significantly increased in the BAL. These factors have been reported during other lung infections suggesting a common defense response to respiratory pathogens. For instance, IL-18 has been shown to activate NK cells and stimulate IFNγ production during influenza infection in the lungs^46^. Increases in IL-6 and IFNγ have also been observed in nasopharyngeal lavage samples taken from influenza patients, BAL samples taken from flu infected rhesus macaques, and BAL samples from rhesus macaques infected with pulmonary nontuberculous mycobacteria^47^,^48^,^49^. The sources of these factors could be alveolar macrophages and lung epithelial cells as previously reported during influenza infection and lung injury^50^,^51^. Indeed, ImmGen analysis also shows IL-18 and IL-1β to be highly expressed by MACs. Interestingly, no chemokines or cytokines were increased in the lung homogenate, suggesting that immune mediators are quickly secreted into the alveolar space and do not remain within the lung parenchyma. In support of this hypothesis, mRNA transcripts of *CXCL9* (*MIG*), *CXCL10*, *CXCL11* (*ITAC*), *IL-1β*, and *IL-8* within lung homogenates were significantly increased. Protein levels of all these chemokines were increased in the BAL at the same time point.

The pDC infiltration 3 DPI correlated with a robust induction of IFNα in the BAL supernatant 3–7 DPI. Another source of IFNα could be epithelial cells, which, like pDCs, express the double stranded DNA sensor TLR9^52^, and could secrete type 1 interferons in a RIG-I dependent manner as described for RSV infection^53^. Alternatively, IFNα could also be secreted by alveolar macrophages, which were the primary producers of IFNα in mice infected with Newcastle disease virus^54^. Concomitant with the increased IFNα levels in the BAL, we detected a significant up-regulation of the transcription factor *STAT1*, induced by type 1 and 2 interferons 3 DPI^11^. *STAT-1* in turn regulates the expression of interferon-stimulated genes (ISGs), which play a critical role in inhibiting viral replication. Indeed, ISGs were among the most highly up-regulated genes in our data set (average FC = 18). The increased expression of ISGs persists until 10 DPI and correlates with decreased viral loads 10 and 14 DPI. Our observations are similar to those described for influenza and RSV where increased expression of *ISG15, RIGI, IRF1, CXCL10, CXCL11, IFIT1* and *STAT1* have been shown to play a role in disease resolution^55^,^56^,^57^. Similarly, reduced ISG expression has been associated with severe cases of H5N1 infection in cynomologous macaques^58^.

*STAT1* also regulates the expression of granzymes (*GRZM*) *A*, *B* and *K*^59^, which were amongst the most highly up-regulated DEGs in our dataset. NK cells and CD8 T cells produce these cytolytic molecules during viral infections to kill infected cells^60^. Increased expression of GRMZ genes correlated with increased frequency of EM CD8 T cells and was confirmed by IHC staining. Bioinformatic analysis using ImmGen also confirmed that GZMA and GZMB was highest in NK and T cells 3–10 DPI. We previously reported increased GRMZB levels within BAL-resident CD4 and CD8 T cells during SVV infection^7^. Elevated *GRZMA* and *GRZMB* expression have also been reported during RSV and influenza A infection^61^,^62^. Moreover, increased expression of GRZMK during acute lung inflammation can in turn induce IL-6, IL-8 and CCL2 production^63^. Both mRNA and protein levels of IL-6 and IL-8 were increased during acute varicella 3 DPI. Together with previous reports, data reported in this study suggest that the lung generates a similar response to respiratory viral pathogens lead by ISGs, granzymes, similar chemokines and cytokines followed by an infiltration of B and T cells.

Acute varicella is accompanied by lung injury as evidenced by the severe focal hemorrhaging and damaged alveolar walls as well as the down-regulation of genes involved in lung development and function. This damage is likely caused by both the anti-viral immune response as well as viral replication as previously described for influenza^64^. Indeed, the damage was most severe at the peak of viral loads and immune response (7 DPI). For instance, expression of *IRX3* (3 DPI), important for lung development and airspace maintenance^12^ and *SEC14-like 3* (7 DPI), which plays a critical role in preventing lung collapse^28^, were significantly down-regulated by SVV infection. Similarly, down-regulation of *TNNT1* (10 DPI) has been proposed as a cause of acute respiratory distress^65^. Moreover, genes that play a role in controlling lung inflammation were down-regulated such as *SCGB3A1* (10 DPI), which regulates airway inflammation and tissue repair^66^. Interestingly, type 1 and 2 interferons, which were increased 3 and 7 DPI, have been shown to decrease *SCGB3A1* expression^67^. Similarly, *IRS2*, which interacts with IL-4 and acts as an anti-inflammatory agent in the lungs^27^, was also down-regulated (7 DPI).

Several tumor suppressor genes were down-regulated, while genes that were involved in the cell cycle were up-regulated, which could have contributed to the increased T cell numbers seen 7–10 DPI. Indeed, analysis using ImmGen indicates that these genes are most highly expressed by T cells. Significant increase in expression of cell cycle genes was also observed in mice recovering from MRSA-induced pneumonia^68^. The up-regulated cell cycle genes could also indicate the beginning of the repair process in the lungs since young immune competent rhesus macaques resolve primary varicella infection without complication. In line with that hypothesis, we detected an increase in growth factors VEGF and PDGF-BB, and angiogenic factor VEGF-A 10 and 14 DPI, which have been shown to play a critical role in alveolarization in the lungs and repairing damaged alveolar walls^69^,^70^. Interestingly, several genes involved with lung function remained down-regulated 10 DPI and 14 DPI, suggesting that repair continues after cessation of viral replication. For instance, KRT5, essential for lung tissue repair^26^, remained one of the most significantly down-regulated genes at 10 DPI, and DNAH2, involved with cilium motility in the lungs^40^ also remained down-regulated at 14 DPI.

In summary, data from this study provides the first kinetic analysis of host-pathogen interactions within the lungs during acute varicella infection. A robust immune response led by interferon-stimulated genes, granzymes, cytokines and chemokines is critical for the resolution of infection. These data also show that although SVV infection in young macaques is self-limiting, significant lung injury occurs during the acute phase as a result of the immune response and viral replication, a phenomenon that has not previously been described. Our results provide potential insights to both what happens in a self –resolving infection, and by extension, what may be happening when infection results in severe complications. Specifically, our RNA sequencing data show reduced expression of genes important for lung function during peak viral replication. In immune deficient individuals who lack the vigorous immune response, uncontrolled viral replication could lead to sustained reduction in the expression of these critical genes, which in turn results in severe lung damage and viral pneumonia. Furthermore, immune genes that are significantly up-regulated likely play a critical role in resolving infection; therefore, lack of increased expression of these genes may explain why the immunocompromised develop severe disease. On the other hand, adults may generate too vigorous of an immune response that results in severe inflammation and viral pneumonia. These data help design interventions to mitigate complications associated with VZV pneumonia and other infectious respiratory diseases.

## Methods

### Animals and sample collection

Fourteen colony-bred Rhesus macaques (Macaca mulatta, RM) 3–5 years of age and of Indian origin were used in these analyses. Animals were housed and handled in accordance with the Oregon National Primate Research Center (ONPRC) Institutional Animal Care and Use Committee (IACUC protocol #0779). The ONPRC Institutional Animal Care Use Committee approved all experimental protocols. The ONPRC has been continuously accredited by the American Association for Accreditation of Laboratory Animal Care since 1974 (PHS/OLAW Animal Welfare Assurance #A3304-01). Eleven RM were inoculated with 4 × 10^5 ^PFU SVV as previously described^6^ and euthanized 3 (n = 3), 7 (n = 3), 10 (n = 2), and 14 (n = 3) days post-infection (DPI); and 3 were controls. Animals were either housed single or paired in caging that allowed for social interactions in a temperature and humidity controlled environment. Food and water were available ad libitum and enrichment was provided daily. All procedures were done under Ketamine anesthesia to minimize animal suffering. Necropsy was carried out in accordance with the recommendation of the American Veterinary Association guidelines for euthanasia. Blood, bronchial alveolar lavage (BAL) and lung tissues were collected at necropsy. Tissues were then flash frozen or stored in trizol at −80°.

### Luminex analysis

Lung tissue was homogenized using 1.0mm Silicon Carbide Beads (BioSpec Products Inc, Bartlesville, OK). Lung homogenate and BAL supernatants were analyzed using the Non-Human Primate ProcartaPLex Cytokine/Chemokine/Growth Factor 37-Panel (eBioscience, Inc, San Diego, CA) which measure the expression levels of: monocyte chemoattractant protein 1 (MCP-1; CCL2), IL-1β, IL-2, IL-10, IL-12p70, IL-4, IL-5, IL-6, IL-7, IL-8, IL-12, IL-13, IL-15, IL-17A, IL-18, IL-23, interferon gamma-induced protein 10 (IP-10; CXCL10), macrophage inflammatory protein 1 alpha (MIP-1α; CCL3) and beta (MIP-1β; CCL4), IL-1 receptor agonist (IL-1RA), TNF-α, Stem Cell Factor (SCF), Eotaxin (CCL11), Fibroblast Growth Factor 2 (FGF-2), B lymphocyte chemoattractant (BLC; CXCL13), Brain-derived neurotrophic factor (BDNF), granulocyte-colony stimulating factor (G-CSF), Granulocyte macrophage colony-stimulating factor (GM-CSF), IFNα, IFNγ, interferon-inducible T cell Alpha chemoattractant (I-TAC; CXCL11), nerve growth factor beta (NGFβ), platelet-derived growth factor-BB (PDGF-BB), soluble CD40 ligand (sCD40L), stromal cell-derived factor 1(SDF-1α; CXCL12a), vascular endothelial growth factor A (VEGF-A) and D (VEGF-D).

### Isolation of immune cells

Lung tissue was digested using 150 U/ml collagenase, 60 U/ml DNAse, 60 U/ml hyaluronidase and 0.2 U/ml elastase for 1 hour at 37 °C with shaking. The digested tissue was then homogenized using a cell strainer to generate a single cell suspension. Cells were isolated using a 30% percoll gradient, then stained with antibodies directed against: CD4 (Tonbo Biosciences, San Diego, CA), CD8β (Beckman Coulter, Brea, CA), CD28 (Tonbo Biosciences), CD95 (BioLegend, San Diego, CA), CCR7 (BD Pharmingen, San Diego, CA) CD20 (Southern Biotech, Birmingham, AL), IgD (Southern Biotech), and CD27 (Biolegend) as previously described^7^. A second sample was stained using antibodies against: CD3 (BD Pharmingen), CD20 (Biolegend), CD14 (BioLegend), HLA-DR (Biolegend), CD11c (Biolegend), and CD123 (Biolegend) as previously described^7^. Samples were analyzed using the LSRII instrument (Becton, Dickinson and Company, San Jose, CA) and Flowjo software (TreeStar, Ashland, OR).

### DNA/RNA Extraction

DNA was extracted from lung tissue using the Qiagen genomic DNA extraction kit (Qiagen, Valencia, CA) and from BAL and blood using the ArchivePure DNA cell/tissue kit (5 PRIME, Gaithersberg, MD). Viral DNA loads were determined exactly as previously described^6^ by real-time PCR using primers and probes specific for ORF21 and ran on the ABI StepOne (Applied Biosystems, Foster City, CA). RNA was extracted from lung tissue homogenized in trizol using a bead beater and zirconia/silica beads followed by extraction using the Ambion Purelink RNA Mini Kit (Life Technologies, Carlsbad, CA).

### Histological and Immunohistochemistry Analysis

Hematoxylin and Eosin (H&E) staining of 5μm lung tissue sections were deparaffinized with Histo-Clear II (National Diagnostics, Atlanta, Georgia) and rehydrated. Tissue was then stained with Hematoxylin for 2 minutes followed by Eosin for 40 seconds and then hydrated back to Histoclear and covered with coverslips using Omnimount (National Diagnostics, Atlanta, Georgia).

For immunohistochemistry analysis, antigen retrieval was done using a pressure cooker in citrate buffer for 20 minutes. Sections were then blocked in 1% bovine serum albumin (BSA) and 5% normal goat or horse serum for 1hr followed by avidin and then biotin for 15 minutes. Tissues were then stained with primary antibodies CD3 (1:200 dilution, Dako M0452, CD20 (1:300 dilution, Dako M0755), CD68 (1:75, Dako, Ki67 1:150 dilution, Dako MIB-1), granzyme B (1:200 dilution, Millipore, Temecula, CA) and VZV glycoprotein B (1:200 dilution, antibodies-online.com clone SG2-2E6). Color development was done with ImmPACT DAB (Vector labs, Burlingame, CA) and counter stained with Hematoxylin QS (Vector Laboratories, Burlingame, CA). Slides were then covered with coverslips using Omnimount (National Diagnostics, Atlanta, Georgia). Images were taken on the Leica DM5500 B (Leica Biosystems, Buffalo Grove, IL) microscope.

### mRNA Library Preparation

One microgram of RNA was used to generate RNA libraries using the using the New England Biolab (NEB) Next Ultra Direction RNA Prep kit for Illumina (Ipswich, MA) and AMPure XP beads (Beckman Coutler, Brea, CA). Each library was made with a unique index primer to allow for multiplexing and then sequenced on the Illumina HiSeq2500 (Illumina, San Diego, CA) platform at single-ends 100bps as previously described^71^.

### RNA-Sequencing analysis

Sequence analysis was performed as previously described^71^. Due to the limited number of animals used in the this study, use used a stringent cutoff with differentially expressed genes (DEGs) being defined as those with a fold change ≥3 and a false discovery rate (FDR) ≤0.05 compared to naïve animals. Enrichment analysis was performed using MetaCore software (GeneGo, Philadelphia, PA). Gene clusters were predicted by STEM using k-means clustering of median normalized reads and maps were generated using ggplot package in R. GEO accession number SRP072466.

### Gene Validation

Gene validation was done using RNA from the same samples used for RNA-Seq. RNA was reverse-transcribed using 100 mM dNTPs (Applied Biosystems) and multiscribe reverse transcriptase (Applied Biosystems) to produce cDNA. Expression was determined using Taqman gene expression assays (Thermo Fisher, Waltham, MA) of BST2, IFITM1, IGJ, MX1 and a housekeeping gene (RPL32). Taqman gene expression assays with 50 ng of cDNA and a housekeeping gene (RPL32) was carried out in duplicates on the ABI StepOne instrument (Applied Biosystems,). Expression of mRNA was normalized to expression levels of RPL32 using ΔCt values as previously described^71^.

### Statistical Analysis

Statistical analysis was conducted using GraphPad Prism software (GraphPad, Software, Inc., La Jolla, CA). Significant values were determined using one-way ANOVA with an alpha value of 0.05 or less.

## Additional Information

**How to cite this article**: Arnold, N. *et al*. Genomic and functional analysis of the host response to acute simian varicella infection in the lung. *Sci. Rep.* **6**, 34164; doi: 10.1038/srep34164 (2016).

## Supplementary Material

Supplementary Figures 1-4

Supplementary Dataset 1

Supplementary Dataset 2

Supplementary Dataset 3

Supplementary Dataset 4
