# Supplementary material for: Genomic and functional analysis of the host response to acute simian varicella infection in the lung
Source: Sci Rep. 2016 Sep 28;6:34164. doi: 10.1038/srep34164 (PMC5039758; doi:10.1038/srep34164)
Supplement: Supplementary Figures 1-4 [file srep34164-s1.pdf]

Genomic and functional analysis of the host response to acute simian varicella infection in the lung

Nicole Arnold[1], Thomas Girke[2], Suhas Sureshchandra[3], Christina Nguyen[4], Maham Rais[4] and Ilhem Messaoudi[1,3,4*]

[1]Graduate Program in Microbiology, University of California-Riverside, CA, USA

[2] Department of Botany and Plant Sciences, University of California-Riverside, CA, USA

[3]Graduate Program in Genetics, Genomics and Bioinformatics, University of California-Riverside, CA, USA

[4]Division of Biomedical Sciences, School of Medicine, University of California-Riverside, Riverside, CA

*To whom correspondence should be addressed

Ilhem Messaoudi, PhD

University of California Riverside

900 University Avenue

Riverside, CA 92521

Tel.: 951-827-7774

E-mail: ilhem.messaoudi@ucr.edu

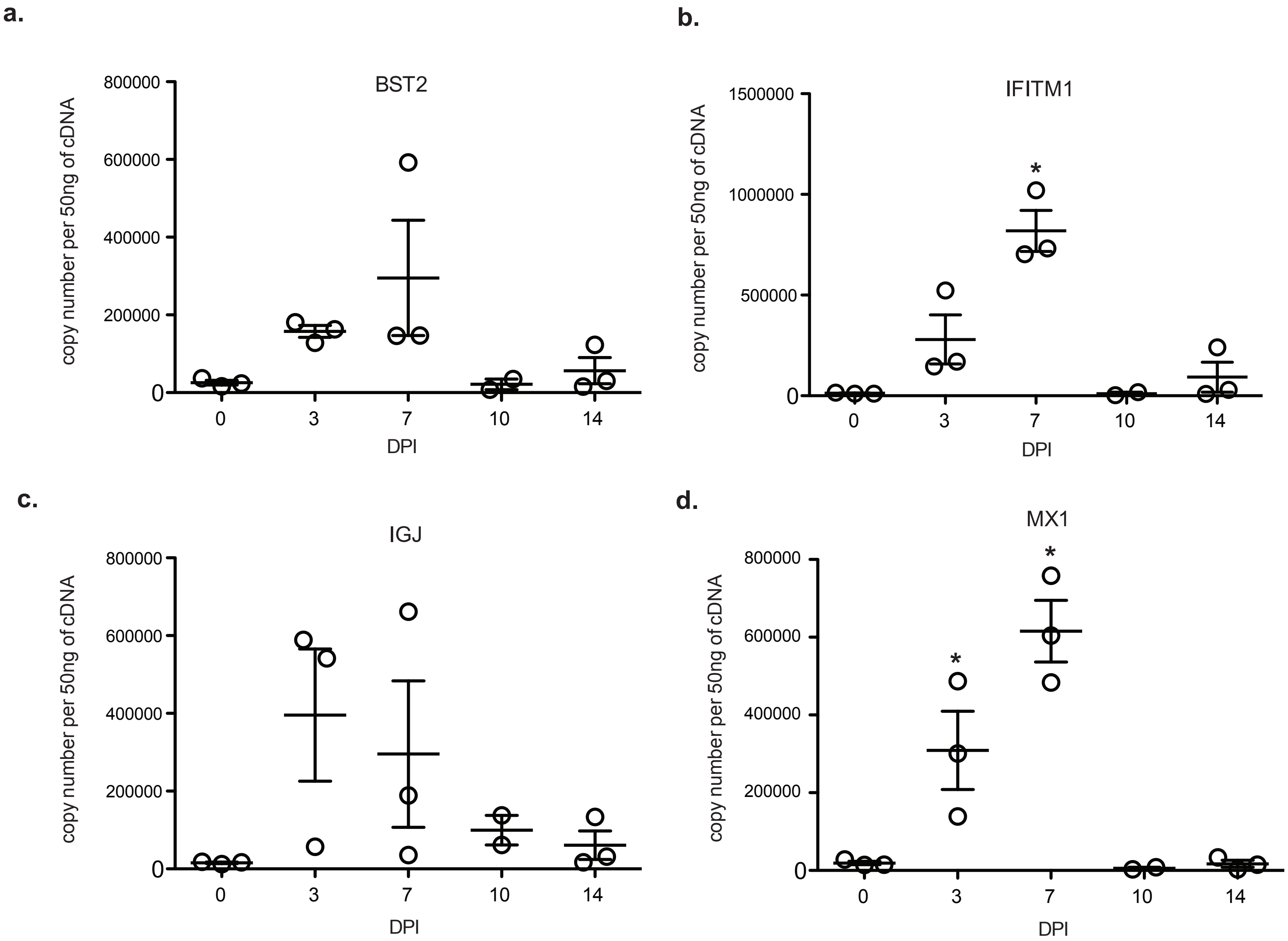

Supplemental Figure 1: Gene Validation. Taqman expression assays were done on (A) BST2 (B) IFITM1 (C) IGJ and (D) MX1. Statistical signficance was determined using one-way ANOVA. Error bars show the standard error of the mean (*, p<0.05 compared to 0 DPI).

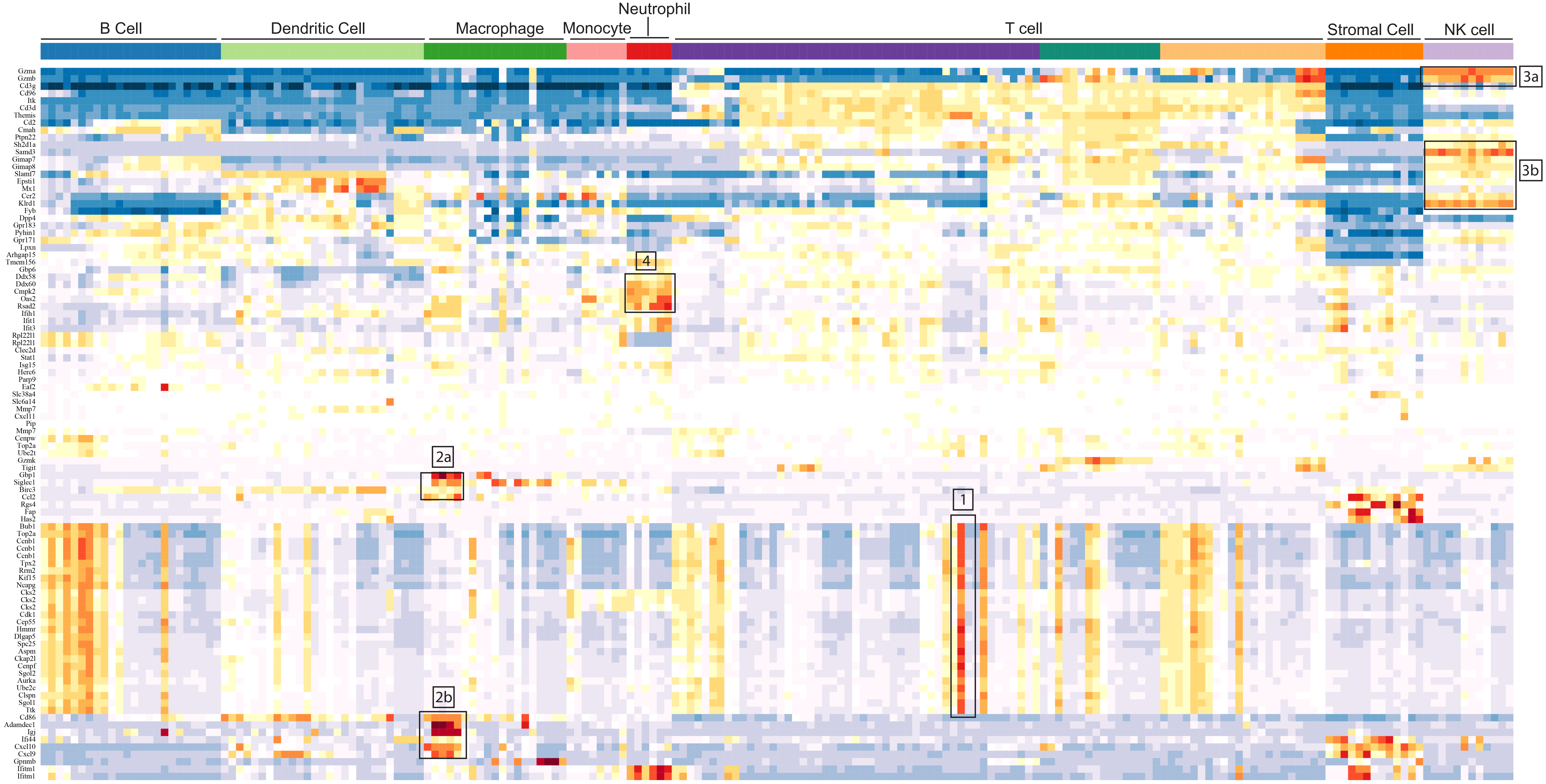

Supplemental Figure 2: ImmGen heatmap showing the expression profile of the genes up-regulated 3 days post infection (DPI) across different immune cell populations. Each row represents a gene and a corresponding microarray ID and columns represents an immune cell type and study. Colors represents expression levels where red indicates the likelihood that the gene of interest is highly expressed by a specific cell type and blue represents low expression. Cluster 1 shows genes involved in the cell cycle (e.g. *Top2a*, *BUB1*, *CEP55*) mapping to B cells, dendritic cells and T cells. Cluster 2a,b shows genes that are highly expressed in macrophages (*ADAMDEC1*, *IgJ*, *IFI44*, *CXCL9* and *CXCL10*). Cluster 3a,b shows genes *SAMD3*, *GZMB* and *GZMA* highly expressed by NK cells. Cluster 4 shows antiviral genes *DDX60*, *OAS2* and *RSAD2* highly expressed by neutrophils.

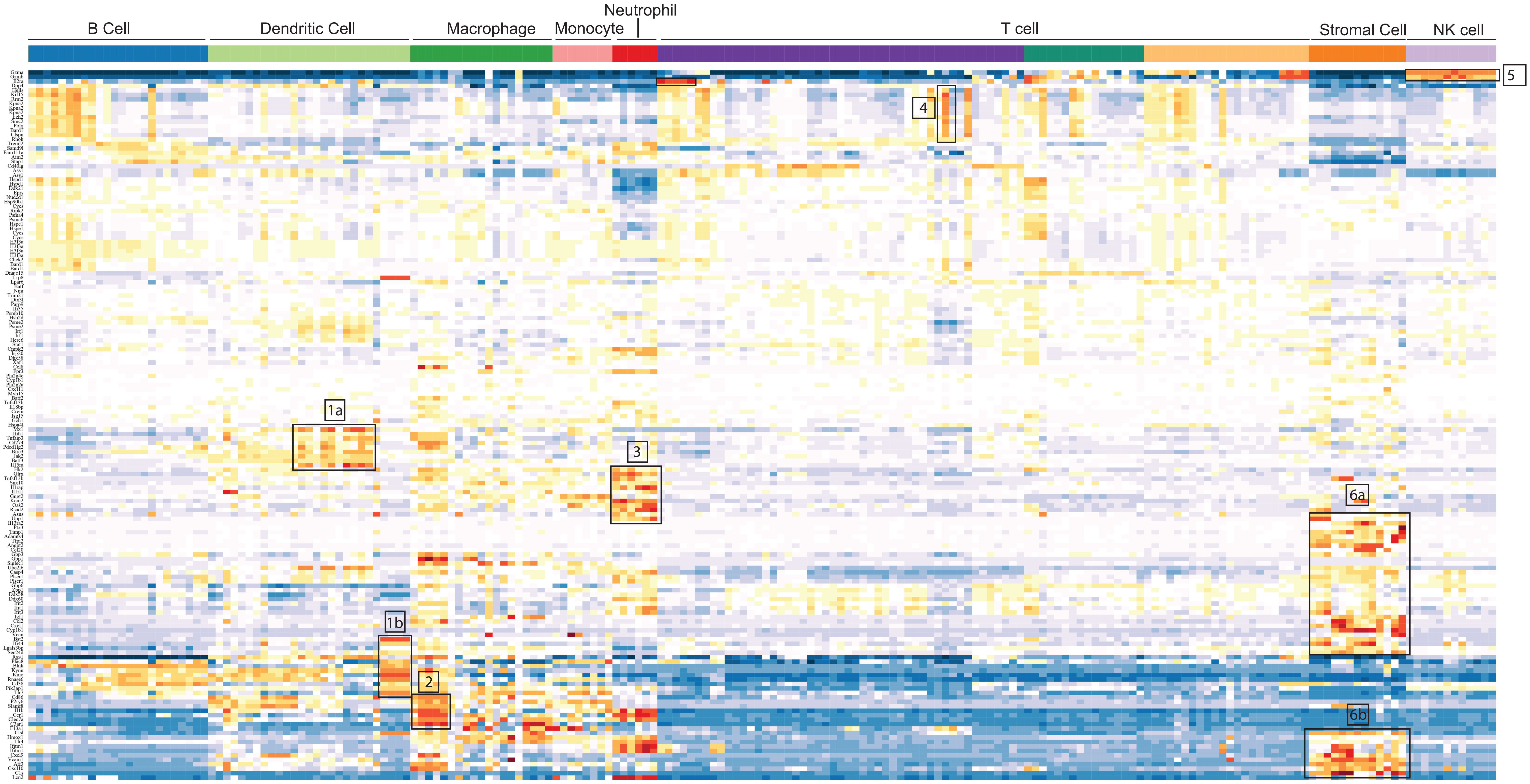

Supplemental Figure 3: ImmGen heatmap showing the expression profile of the immune genes up-regulated 7 DPI across different immune cell populations. Each row represents a gene and a corresponding microarray ID and columns represents an immune cell type and study. Colors represents expression levels where red indicates the likelihood that the gene of interest is highly expressed by a specific cell type and blue represents low expression. Gene clusters 1a,b show antiviral genes (*MX1* and *IFIH1*), immune homeostasis genes (*CD274* and *PDCD1LG2*) and genes involved in apoptosis (*TNFAIP3* and *BIRC3*) highly expressed by DCs. Cluster 2 highlights a cluster of genes highly expressed in the macrophages that are involved in the proinflammatory response such as *CD86*, *IL-1β* and *CCR1.* Cluster 3 shows antiviral genes (*OAS2* and *RSAD2*) and proinflammatory genes (*TNFSF13B* and *IL1RAP*) primarily expressed in neutrophils. Cluster 4 shows highly expressed genes shared by B and T cells (e.g. *RRM2, KPNA1* and *EZH2*). Cluster 5 shows genes *GZMA* and *GZMB* are highly expressed by NK and T cells. Clusters 6a,b shows genes highly expressed by stromal cells, many of which are also involved in proinflammation (e.g. *CCL1, CXCL1, CXCL9*, *CXCL10* and *IFITM1*).

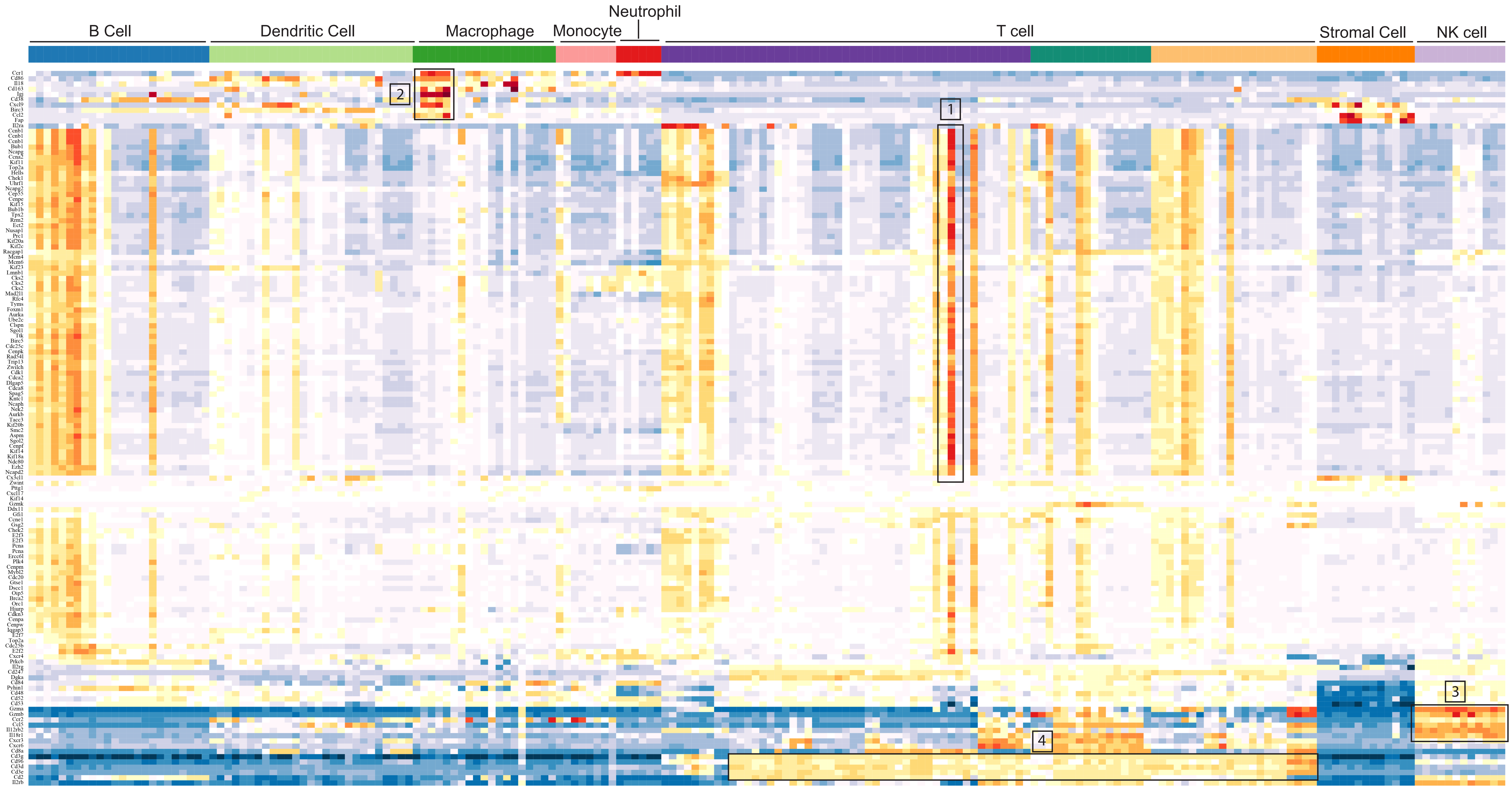

Supplemental Figure 4: ImmGen heatmap showing the expression profile of the genes up-regulated 10 DPI across different immune cell populations. Each row represents a gene and a corresponding microarray ID and columns represents an immune cell type and study. Colors represents expression levels where red is indicates the likelihood that the gene of interest is highly expressed by a specific cell type and blue represents low expression. Cluster 1 shows a large number of genes involved with the cell cycle (e.g. *CCNB1, KIF11, TOP2A, SPAG5* and *CENPF)* that are highly expressed by T cells and B cells. Cluster 2 shows genes highly expressed by macrophages (*CD163*, *IL-18*, *CCR1* and *CXCL9)*. Cluster 3 shows genes that are highly expressed by NK cells and T cells (*GZMA, GZMB, CCR2* and *CCL5*). Cluster 4 shows genes highly expressed by T cells (*CD96, CD3D,* and *CD2).*
